# Report on Surgery for the Semi-Annual Session of the Æsculapian Society of the Wabash Valley, May 25, 1871

**Published:** 1871-09

**Authors:** Leon J. Willien

**Affiliations:** Effingham, Illinois


### REPORT ON SURGERY

*For the Semi-Annual Session of the Æsculapian Society of the Wabash Valley, May* 25, 1871.

By Leon J. Willien, M.D., Effingham, Illinois.

*Gentlemen, Members of the Æsculapian Society:*

> Doctrines pass away, and facts remain,—
> So it is said. Error?
> When doctrines pass, the facts are forgotten.—*Gubler.*

> And if the art of healing is, of all practical sciences, the most difficult, should we not, perhaps, do all that is in our power to remove the difficulty? Although the method is very easy, it would be to the restriction of observation only.—*Marjolin.*

The duty of making a report on surgery being allotted to us, although this great branch should be wielded by an older and more experienced member of this Society—but, *si qua fata sinant*, we shall give you the best our short experience can afford.

It is not our object to make a retrospective history of surgery, nor shall we act as a critic on late discoveries of the new anæsthetics which are to substitute the one which we consider the most dangerous—chloroform.

The hydrate of chloral, which has been used with such great success of late years, has but recently been looked upon as of a dangerous administration, on account of the uncertainty of its effects on the system, and no antidote having been discovered until a short time since by Dr. Oscar Liebriech, who shortens and illuminates its effects by using the injections with the nitrate of strychnia.

In England, bichloride of methyline has the sway, and, latest, we see methyle ether used as a general anæsthetic. These will be sufficiently commended and criticised in our numerous medical journals. We will, therefore, not burden your attention with unfounded theories and hypotheses, which are only well appropriate to authors in order to puzzle the younger members of our profession, and cause the older ones to exclaim with a sigh, "What new discoveries next!"

No communications having been sent us from any of the Committee on Surgery, this report will only be a short review of personal experience at home. We, therefore, hope that this short report will be received with indulgence, and the little merit it possesses will be appreciated.

We shall now introduce the subject of the influence of fractures of limbs on the growth of the nails.

Several articles have been published in the London "Lancet" for March, 1869, page 149, by Dr. Wilkes, on markings and furrows on the nails as the result of illness; and in the April number tor 1870, page 210, we find a second article by the same author on the furrows on the nails *after* illness. These, however, are the results of medical observations, while we shall look on the subject in a surgical point of view. In 1866, our attention was first called to it in our practice, in giving aid to a boy eight years of age, having a fracture of the humerus. His finger-nails were stained at the same time with dye. We perchance discovered that the nails of the sound arm continued growing, while the one on the other limb only began to grow about the fourteenth day. We then began to experiment on other cases of fractures, to convince ourselves of our first observation. We consulted different American and foreign authors and journals, without finding anything written on the above statement. At last we found an article, published in the French "Medical and Surgical Journal," of Paris, for 1868, volume 39, page 215, on the semiotic value of the growth of the nails in fractured limbs. In 1842, Dr. Guenther, from Denmark, made mention of the nails as a sure means of recognizing the consolidation of fractured bones. According to his statement and our own observation, the growth of the nail ceases as soon as a solution of continuity exists in the shaft of a bone; and this cessation of their growth is already a sign of a fracture, and in growing again, after a certain length of time, becomes a still more certain indication, because it shows that the consolidation of the bone is affecting itself regularly.

Was it an unfounded theory of ours, or was it Dr. Guenther's fictitious imagination? Or was he the dupe of his patients in believing this curious assertion in regard to the cessation of the growth of the nails in the fracture of one or more bones of a limb? The great critic, Malgaigne, declared it; but if we give credit to Dr. Louis Ansel, author of a monograph on the nails in an anatomical, physiological and pathological point of view, then our renowned surgeon has surely been too hasty in annulling Dr. Guenther's observation. Dr. Broca has several times approved of the veracity of the statement of the Danish physician, by a patient who had a fracture of the tibia a few centimetres above the tibio tarsal articulation. During the whole time preceding the process of consolidation, Dr. Broca marked the toe-nails of both feet with lunar caustic, and discovered very plainly that the growth of the nails on the fractured limb had ceased, while the ones on the other foot continued growing.

Dr. Duplay, of Paris, makes mention of a case where there was a fracture of the left fore-arm, accompanied by a disease, retarding the consolidation. The accident happened on the 7th of October, 1867, and only on the 19th of November was he able to apply a starched bandage, and prescribed forty grains of phosphate of lime daily.

At this time, the patient called his attention to the state of the finger-nails of the injured arm, these having ceased growing since the 7th day of October, and having a dark-yellow tinge. A few days after the application of the splint and the taking of the phosphate of lime, these began to grow, and a little red ring was seen.

From that time, their growth proceeded rapidly, and a well-defined lineament was seen between the old and new nail, and on the 31st of December, there was scarcely more than one-fourth of an inch left of the old nail. On the 10th of January, the ulna was completely solidified. But an incident worthy of notice: Consecutive to fatigue, the consolidation of the radius stops, and remains stationary during fifteen days, and immediately the growth of the nails cease, a ridge being visible at the external surface. Consolidation resumed its work, and at the same time, a positive and regular growth of the nails was again visible.

In presence of these facts, and others which we shall mention, it can not be denied that the trouble of nutrition, which strikes the broken bones of a limb, has also its influence on the growth of the nails where the unequal secretion is in near connection with the process of consolidation of the fractured bones. No member of this Association will deny that this sign is of great importance to all surgeons, especially in cases of pseudarthrosis, where direct and repeated examinations are often too prejudicial to the patient; also, in cases of necrosis and in fractures of the neck of the femur.

We will notice a case of circular saw injury to corroborate our remarks on the growth of nails.

Mortimer F——, aged thirty years, while feeling the temper of the saw while in motion, was seized by the teeth, which produced the following disorders of the hand and arm:

First—A complete external dislocation of the elbow.

Second—A ghastly and transverse wound extending from the first to the fourth metacarpal bones, severing the tendons of the dorsal part of the hand sectioning the extensors of both annular and medius, extending through the articulation of the second and third carpo-metacarpal, tearing their ligaments and injuring the deep palmar arch; also, a deep wound of the phalingo-metacarpal joint of the index, severing the interosseous artery.

At our arrival, the patient was sinking fast on account of severe hemorrhage. We proceeded immediately to compress the humeral artery with the tourniquet, and began to dress the wounds, all contused and lacerated tissue, with small fragments of bones (or parcels), being removed. Yet, it was not possible for us to reach the artery without enlarging the wound. The palmar portion of the hand was supported by a splint, the wound then plugged with lint, imbibed in a strong solution of persulphate of iron and fluid extract of ergot, and then the whole was firmly bandaged, keeping the bands saturated with the following embrocation: ℞—Carbolic acid, f ʒ ss; tinct. arnica, f ℥ iv; and ordering pieces of ice to be maintained on the hand. Gradual compression of the humeral artery was continued, and the luxation of the elbow was easily reduced and the arm bandaged. On the third day, the first bandage was removed, and gradual compression of the artery continued. The wound was cleanly washed and dressed with a mixture of carbolic acid ten grains to glycerine one ounce; no other application was made. The nails of the index medius and annular ceased growing until the fourth week after the accident, when they resumed their growth again. On the 15th day of January, the patient was discharged.

While attending the above case, we were called to a man twenty-seven years of age, having a fracture of the neck of the femur, and on his foot the nails only resumed their growth on the eighteenth day after the fracture.

We will now come to the second and last part of our report, in which we will relate some of the most interesting cases in our own practice.

A CASE OF IRREDUCIBLE STRANGULATED CRURAL HERNIA.

Mrs. D. P——, aged thirty-nine years, mother of five children, affected with a small irreducible hernia of ten years' standing, resident of Effingham, was suddenly taken ill in July, 1869, with all the symptoms of strangulation of the bowels, constipation, meteorism, and severe pain in the region of the hernia, which was situated in the right crural region.

After practicing taxis and administering the usual remedies, both local and internally, without relief, the general symptoms increased in gravity, with stercoraceous and bilious vomiting. Being called in consultation with Drs. Secrone and St. Clair, we at once decided to have recourse to celotomy as the only means to give the patient a chance of recovery. On the 7th day of July, at six o'clock P.M., the patient was laid on the table and chloroformed. The tumor was about the size of a large hen's egg, elastic and slightly inflamed, very painful on pressure. We made an incision parallel to the larger diameter of the tumor, comprising the skin, then the fascia superficialis which was adherent to the fascia propria; here there was a complete adherence of the cellulo-adipose tissue with the sac, which had to be separated with the finger. The sac being exposed, we found it of a dark-brown color, and mortified, causing very fetid exhalations; and upon opening, we found its inner portion adherent to a portion of the strangulated bowel, for the sac contained no serosity.

After exposing a portion of the strangulated portion of the intestine, which was about the size of a large walnut, we removed all adherence, by aid of the finger, until we reached the stricture. Here, around the neck of the sac, there were fibrous adhesions, which were removed with difficulty. The stricture was so strong as not to allow us to pass the blunt and thin edge of the cannula. We then slipped the index slowly and firmly between the bowel and the fallopian ligament, slipping along its side the blunt bistoury of Cooper, making two free incisions, one outward, which suffices to relieve the bowel. No signs of gangrene being discovered, and convinced that all obstruction was removed, we reduced the bowel and excised the mortified portion of the sac. Very little blood was lost. The wound was well sponged, and the incision closed with three sutures of silk thread, and carefully bandaged. Ice was applied to the wound, and opium given internally to quiet the bowels and relieve pain.

On the third day, suppuration began, yet the bowels were highly meteorized; but there were otherwise no alarming symptoms. The wound was dressed with simple cerate and carbolic acid during the first three or four days, after which carbolized glycerine was used.

On the seventh day, after giving a dose of oil, the bowels were evacuated freely, accompanied with pain; the flatus ceased, the wound healed well, the appetite returned, and with it strength, and once more was the patient in a condition to enjoy health, having been radically cured. We will remark, that the patient was pregnant, about six weeks advanced, at the time of the operation.

PERI-UTERINE ABSCESS—SUPPOSED CAUSE, PERIMETRITIS.

Mrs. A. H———, aged twenty-seven years, mother of two children, residing in Effingham, in the year 1866, while making an effort to lift a tub, was seized with severe pain in the back, followed by severe uterine hemorrhage, so that it was difficult for the medical attendant to recall her to consciousness. She being at the same time pregnant, contraction soon set in; miscarriage of fetus of four months was the consequence. From that time, she continued to suffer with pains in the lower part of the abdomen—these darting to and fro to the sacro-lumbar regions, with frequent strangury and tenesmus of the bowels.

A physician from Decatur having been consulted, he pronounced said disease to arise from a uterine polypus, and she immediately demanded its removal. After being chloroformed, heavy tractions were made upon the neck of the womb in order to bring it down close to the vulva. But where was the polypus then? The operator did not reach it, and the operation was abandoned by saying that "it could only be reached and removed by escharotics." The fact was, there was no polypus. The patient's sufferings began to increase from that time.

In December, 1867, her health became better, and she menstruated regularly. These periods were heretofore painful and irregular. She soon became pregnant, and again miscarried at the fourth month; and after recovering from her puerperal condition, she moved to Effingham. Here she again took sick, her symptoms aggravating more and more until the latter part of February, 1869, when we were called to visit her.

The patient presented the following symptoms: Decubitus dorsal, and face pale with expression of pain and anxiety; anorexia; pulse frequent and weak; chilling frequently, and followed during the night with cold and abundant perspiration, severe pains through the abdomen, constipation, vesical tenesmus, and severe pains through the lower part of the abdomen.

On palpation, we found a tumor of the size of a large infant's head, extending above the pubis about three inches. This tumor was painful on pressure, distended and fluctuating, movable to a small extent right to left, but not anteriorly or backward nor upward or downward. Per vaginal exploration, the cervix was inclined to the right, its posterior labiæ being enlarged, leaving little of the retro-vaginal cul-de-sac. The os was partly open and granulated, as was discovered by speculum, from which exudated a sero-sanguinolous liquid of very offensive odor; besides, the uterus was considerably lowered. In the cul-de-sac, fluctuation was evident, and the tissues considerably enlarged. By exploration per rectum, the tumor was easily felt. But was this an intra-uterine disease, or its tissue proper undergoing some malignant degenerescence, or perhaps a polypus in utero or a peri-uterine abscess? The probe of Belloc was introduced into the uterus, which showed a deviation of that organ to the left. Sponge tents were introduced into the os in order to dilate it at the neck. We then explored its cavity, and found it exempt from any disease or tumor. There was, therefore, no doubt left in our mind that it was a liquid, which had accumulated between the peritoneal membrane and the tissue proper of the womb. But was this liquid serous, blood, or pus? The general symptoms and anterior history of the patient gave us sufficient reason to suspect the formation of pus, which had been formed consecutive to peri-metritis. We were impatient to see the issue, and have our client relieved from pain. After a careful consideration, and at the urgent request of the sufferer, we decided to operate.

On the 30th of March, 1869, the patient being placed under the influence of chloroform by Dr. Secrone, we plunged a small trocar through the vaginal cul-de-sac into the fluctuating tumor, and to our satisfaction, there issued through the cannula a thick jet of white inodorous pus, and the tumor decreased. We then pushed an injection, with carbolic acid and tepid rain water into the cavity, and let it sojourn about ten minutes and again allowed to escape, and the cannula was withdrawn. Narcotic poultices were applied on the abdomen, and opiates inwardly to relieve pain; besides sulphate of quinia also freely given, with other tonic and martial preparations and a good diet.

On the fourth day the tumor began to enlarge again, notwithstanding the free evacuation of pus through the vagina. On making an exploration per rectum, the pressure of the finger ruptured the membrane, and the pus was freely discharged. Although the patient was in a despairing condition, the discharges became less abundant. Strength gradually increased, with good appetite. After several months' duration, she was able to walk about and do her work.

### DOUBLE AND COMPLETE FRACTURE OF THE LOWER JAW.

Charles B——, aged thirty-two years, of a robust constitution, a blacksmith by profession, while attempting to bridle a horse, was kicked directly under the left and anterior side of the lower jaw, fracturing it in two places: First—a complete and transverse fracture between the two last molar teeth, with considerable laceration and contusion of the surrounding tissue, a small portion of the upper fragment protruding through the skin; second—a complete and transverse fracture between the cuspidatus and lateral incisor teeth.

None of the teeth were displaced excepting the wisdom tooth, which was loosened. The patient was much exhausted, in consequence of loss of blood and being jolted in a wagon over rough roads for a distance of fifteen miles. The fractures were readjusted as well as the circumstances would permit on account of the œdema of the tissues, and the fragments were maintained in place by slipping a silver wire between the two incisors and the second bicuspid and molar, and twisting them close together, a frond placed around the chin in order to hold the fracture in place; astringent gargles used freely, and sulphate of morphine internally to relieve pain. The swelling having decreased, on the third day, we proceeded to apply a permanent splint. We succeeded admirably in this, by the ingenious hand of our dental surgeon, Dr. L. P. Besier. After the removal of the bandages, the parts were held in place by silver wires, while Dr. Besier took an impression on wax, and made a splint of vulcanized rubber, extending from the wisdom to the canine tooth included. This splint fitted the case *a merveille*. After stimulating the patient with brandy, his mouth was kept open by an assistant; a silver wire was passed between the two first molars, and slipped through the upper aperture of the splint.

During traction, by a sudden motion of the patient, the wires applied on the anterior fracture broke; this enabled us to coapt the posture fracture first, by pressing the splint firmly over the teeth. The anterior fragment was then brought in place, and the splint was fastened down with wires in the interstices of the teeth, and fastened over the splint, rendering the whole immovable.

The plate fitted over the wisdom-tooth with a thick prominence over it, in order that the upper tooth might press on it, and keep the posterior fragment in place. The jaw was then supported from below by a semi-lunar pine-board, with a strip of tin at the anterior edge, well packed with cotton, in order to prevent the pressure or irritation of the thyroid cartilage during deglutition. This was fastened to the crown of a hat without rim, with elastic bands. Low diet and rest was ordered during six weeks, at the end of which he returned to Effingham to have the splint removed. The whole was a perfect success—a moment of joy for our patient, and a feeling of satisfaction for us in seeing the happy result.

The case which we have reserved for the close of our report will, perhaps, be entirely new to most of the gentlemen present. It is of such an interesting character, that we might doubt the veracity of the person relating it. Yet we find new diseases every day, especially in surgery. An artificial or accidental anus is mostly found communicating with the vagina, bladder, right or left hypochondriac regions, inguinal, crural, or where the bowels have nearest egress through canals or thinness of tissue, and most of these are established by surgery or traumatism. This case is an exceptional one, and full of interest.

### ARTIFICIAL ANUS NEAR THE LEFT TROCHANTER—RECOVERY.

Andrew Wheelan, aged forty-three years, tall and strongly constituted, foreman of track-layers on the St. Louis, Vandalia and Terre Haute Railroad, had never been sick in bed, and enjoyed good health until the latter part of October, 1869. A few months preceding this time, he felt more or less pain in the lower part of the abdomen, but it did not prevent him from work. He was often affected with diarrhea, and mucosanguinous discharges. The first symptoms were chilly sensations, with lassitude, extending along the spine and increasing along the thighs and lower part of the abdomen. Fever then set in, and he was obliged to seek relief. * * *

We are not able to give a definite history of the symptoms in the beginning of his illness. We visited him for the first time on January 16, 1870. We found the patient in the following condition: Great emaciation, dyspnœa; eyes sunken, with pale expression; face is pale yellow color, covered with abundant perspiration, with an expression of pain and anxiety; cold sweats would appear and disappear at short intervals, and assisted in dragging down the little vital power left; tongue hard and dry; pulse small, weak, one hundred and twenty per minute; frequent mucous eructations and belching offensive gas; tympanitis considerable, showing the circumlocutions of the bowels; severe pain in the left hip, with an impossibility to move the joint upward or downward; palpation very painful just backward and a little below the trochanter major, with enlargement of the tissues, with a crepitant fluctuation in the region extending from about the upper and outer third of the thigh upward along the pyriformis muscle.

We, therefore, suspected an abscess by migration, which then accounted for the above pyæmic symptoms.

Ordered narcotized poultices to the hips and tonic preparations inwardly, such as sulphate of quinæ, elixir, protoxide of iron, and Peruvian bark and wines, with a good diet.

*January* 17.—Symptoms ut supra. After ascertaining the most superficial point of the abscess, we made a free incision about one inch below and one half inch posteriorly of the great trochanter; a large quantity of dark fetid pus, mixed with bubbles of gas, escaped from the opening. The patient felt relieved, and the general symytoms began to improve, excepting the meteorism, which continued; also the eructations. Poultices sprinkled with laudanum and same treatment.

*January* 18.—The wound discharged profusely during the night. Pulse eighty-nine—regular, but weak; bowels have not been evacuated for three days; other symptoms ut supra. Ordered an injection with castile soap and assafœtida—to be repeated, if necessary; morphine in case of restlessness.

Six P.M.—Two injections have been given, and an abundant evacuation of hard stercoraceous matter was obtained. The nurse insisted at the same time that stercoral substance passed through the incision. Same treatment, and a carminative liniment to be frictioned over the bowels; carbonate of magnesia internally, with milk to relieve these eructations.

*January* 19.—Eructations less frequent, and flatus of the bowels much decreased. There was a great amount of gas and stercoral substance discharged from the incision, the bowels evacuating freely at the same time.

While examining the wound, there was a rush of very offensive gas and a thick chymy liquid and stercoral matters evacuated. This attracted our attention and struck us with astonishment. On palpation, the passage of this liquid and gases was traced up along the pyriformis muscle, and gave us all reason to believe it came through the ischialic foramen, the bowel being perforated at or near the sigmoid flexure of the colon.

No special treatment was used, and all we could do in a case of this kind was to help the strength of the poor sufferer, and leave the other to Providence and efforts of nature. Carbolic acid was injected, with tepid rain water as an antiseptic, and tonics, iron preparations and wine, with full diet, were continued.

*March* 1.—Andrew has good appetite, sleeps well, and is gaining strength fast. The bad sores have healed, and the wound suppurates slightly, leaving no stercoraceous substance through. The bowels are regular.

*May* 1.—The man is again at work on the railroad, entirely well.—*Cincinnati Lancet and Observer.*

